# Construction of Sports Training Performance Prediction Model Based on a Generative Adversarial Deep Neural Network Algorithm

**DOI:** 10.1155/2022/1211238

**Published:** 2022-05-21

**Authors:** Gang Li

**Affiliations:** Physical Education Department, Shenyang Institute of Engineering, Shenyang, Liaoning 110136, China

## Abstract

The generative adversarial neural network algorithm is used for in-depth research and analysis of sports training performance prediction, and the corresponding model is built and used for practical applications. To address the problems of gradient disappearance, training instability, lack of local consistency of repair results, and long training time in the image restoration algorithm based on generative adversarial networks, this paper proposes a multigenerative adversarial image restoration algorithm based on multigranularity reconstruction sampling. The algorithm changes the distribution initialization of the generative network and uses reconstruction sampling to ensure that the Lebesgue measure of the overlapping part of the generative sample space and the real sample space is not 0 to further stabilize the gradient, and it is demonstrated that reconstruction sampling can stabilize the training and gradient. In addition, segmentation invariance is used to shorten the training time while ensuring the quality of the restored images, and an algorithm adaptability metric is proposed to comprehensively evaluate the image restoration algorithm. Based on the results of the fusion model analysis, an attention-based mechanism for the student performance prediction model is proposed. First, deep student behavioral features are extracted using a generative adversarial deep neural network, and the salient features in the student behavioral features are selected using a maximum pooling method; then, the extracted features are used as the input of the generative adversarial deep neural network for student performance prediction. Finally, a temporal attention mechanism is introduced at the output of the generative adversarial deep neural network to assign attention weights to different weekly student behavioral features.

## 1. Introduction

In recent years, information technology has been developing, and the introduction of information technology into university management systems has become a popular research area. The information technology construction has influenced the education of colleges and universities from all aspects, and there are great changes in the content and form [1]. Through the establishment of an “intelligent campus” of education informatization, the management level and service quality of colleges and universities are improved, and an open, collaborative, and intelligent information service platform is formed. The normal operation of the digital and intelligent campus construction system of colleges and universities has accumulated a large amount of campus data and formed the big data environment of colleges and universities. The construction of a big data platform realizes the storage, modeling, and standardized use of college data, and how to use these big data to solve the practical problems of colleges and universities is the core problem of establishing an education big data platform. There is no significant shortening or elongation of the muscles and no significant movement of the joints during the exercise [2]. Predicted outcomes are also categories of students' final exam scores. The model based on a supervised learning algorithm refers to training through the existing training sample set, finding the optimal prediction model suitable for the data set, and then using this optimal prediction model to predict the test set. However, in training, to complete the full-amplitude movement, the load can only be set at the minimum muscle moment.

Therefore, most of the time resistance moment in the movement is slower than the maximum muscle moment, and the exercise effect is somewhat affected [3]. The types of sports that are widely respected in the society now include competitive, recreational, popular, and medical sports: competitive sports are the objective, reasonable, and all-round training and competition that people want to beat their opponents and get good sports results and greatly cultivate and strengthen themselves and their groups in terms of body type, physical strength, emotions, and another biased will; recreational sports are the sports that people carry out in their free time or in designated places to achieve the joy of physical fitness [4]. Recreational sports are activities that people can carry out in their free time or in designated places to achieve effect happiness; such activities have the characteristics of nonprofessional, leisure, pleasure, and so on and are usually held in ball games, chess and card games, trips, some streams of national activities, and so on; popular sports are sports activities that people want to improve physical fitness, fight diseases, train elite candidates, and spend time in real life.

Traditional physical education adopted statistical tools, often taking Excel office software and comprehensive class education office system; the main shortcomings of such tools are manifested in the manual input method work intensity, complex and inefficient form stacking, weak analysis, and poor data readability. For administrators and teachers, it not only increases the daily workload but also makes it difficult to process and analyze the data. To input this new set of sample values into each decision tree of the random forest, these decision trees judge the eigenvalues of these samples, respectively, thereby determining the predicted value under the sample value. For students, it is impossible to get real-time and effective feedback from daily teaching activities and physical fitness tests. In terms of analysis methods, the analysis methods used by the above tools remain on top of simple variance, mean, and reliability calculations, resulting in the conclusions obtained from the analysis staying on the surface and failing to bring the full value of a large amount of data into play. However, what administrators, teachers, and students care about is the hidden information that is not so easy to find. In this paper, we try to use data mining techniques to study and analyze the physical fitness data of college students and to mine and analyze the information related to college physical fitness to get more valuable hidden information. This will help students understand their physical fitness status more clearly and help teachers to start teaching activities in time. This will make the physical education work of universities more timely, effective, and relevant, improve the quality of physical education in universities, and help students improve their physical fitness level and develop good exercise habits.

## 2. Related Works

Sports data are rarely applied to good data mining and feature selection techniques and even less applied to study the impact of sports on physical index data [5]. In related research at home and abroad, the ID3 algorithm, an algorithm of decision tree analysis, is applied to study human grip muscle strength test data, and root nodes of different test index parameters are determined to get the indexes that can scientifically evaluate human muscle strength. An optimized random forest algorithm is proposed to optimize the classifier using an artificial bee colony, and the model recognizes human movement patterns, which can obtain a relatively high classification accuracy [6]. In addition, some works use statistical methods for the study of the effect of sports data on physical fitness. Most of the research on feature selection algorithms has focused on supervised feature selection, which uses category information as a priori to select the subset of features that best distinguish between categories by measuring the interrelationship between features and categories [7]. Unsupervised feature selection refers to feature selection by grouping feature dataset samples using clustering or unsupervised learning methods to evaluate the importance of features without the guidance of category information [8].

Based on the presence of different numbers of categories, feature selection algorithms can be classified into multicategory feature selection and binary feature selection. When a sample data for feature selection contains information from more than one category, it is called multicategory feature selection, also known as multilabel feature selection [9]. According to the different organization of categories, the multicategory problem can be shown as two structures, tiling structure and hierarchical structure [10]. The relationship between categories in the tiled structure is equal, and if the relationship between categories is not independent but has some complex relationship, then it can be processed using hierarchical feature selection [11]. The actual operations mainly include deleting duplicate data, supplementing incomplete data, and correcting erroneous data, so that the cleaned data is standard, clean, and continuous and meets the requirements of subsequent calculations. Data simplification, deduplication, and standard formatting are accomplished through data cleaning. Because of the widespread use of campus card (one card) in universities, domestic scholars' work on education data mining is based on the study of students' campus card consumption behavior [12]. The consumption quantity and the consumption amount were analyzed, the data were equidistantly discretized, and the discrete data were clustered by using Korhonen neural network to provide a reference for decision-making in universities [13].

Considering the relationship between source and target domains, a new sparse learning model is proposed based on an unsupervised adaptive domain, which can jointly reduce the domain differences, find the most discriminative features, and then perform classification. Semisupervised feature selection is accomplished by using a small number of samples with supervised information and many samples with no category information for classification [14]. An expert hybrid classification learning algorithm based on labeled and unlabeled data training is proposed to improve the performance using unlabeled information. Semisupervised feature selection is based on semisupervised learning for feature selection, and with the development of semisupervised techniques, the study of semisupervised feature selection has received increasing attention. In this paper, we propose reconstructive sampling to avoid the overlapping part of the real image distribution and the restored image distribution with a Lebesgue measure of 0 to stabilize the gradient of the generative network and the training process and further achieve high-quality image restoration. This in turn affects the results of data processing. The performance category is used as the category output to train the above four models, and the fivefold cross-validation method is used to test the effect of each classification model. To evaluate the effectiveness of the performance prediction model, the accuracy, precision, recall, and F1 value are used.

## 3. Generative Adversarial Deep Neural Network Algorithm Design

The explosive growth of multimedia data and the rapid development of social media in the past decade have attracted increased researchers to study large-scale image retrieval. In this research, in addition to the traditional content-based retrieval methods, hash retrieval methods have received increasing attention from scholars. Hash algorithms use hash functions to map high-dimensional images into compact binary hash codes and then achieve fast and efficient retrieval by calculating the Hamming distance between the hash codes. At this stage, a series of deep learning-based image hashing methods have been proposed by using deep neural networks to learn both feature representations and hash codes [15]. In addition, generative adversarial networks have also been used to further improve the performance of deep hashing.

To achieve effective retrieval of incomplete images, this paper embeds feature recovery into image hashing retrieval and proposes a noncomplete image hashing retrieval model, consisting of three parts: generative network, discriminative network, and hashing network. There is almost no drop in accuracy, which verifies that BH2I-GAN can concentrate sample points of similar data points within a radius of 2 while moving sample points of dissimilar data to regions with a radius greater than 2. The generative network is used to recover the missing features of the incomplete image, the discriminative network is used to distinguish the output image from the real image or the recovered image and determine whether the two images are similar, and the hash network encodes the incomplete image and the complete image into a compact binary hash code.

(1) $$\begin{array}{c} y_{i}=\beta_{0}-\beta_{1}x_{i1}-\beta_{2}x_{i2}-,\ldots ,-\beta_{p}x_{ip}. \end{array}$$

The coding loss is used to maintain similarity between similar images and nonsimilarity between nonsimilar images, and the quantization loss controls the loss caused by relaxing continuous features into discrete hash codes.

(2) $$\begin{array}{c} \log\;P\left(H|S\right)\infty \;\log\;P\left(S|H\right)\log\;P\left(H\right)=\underset{s_{ij}}{\sum }\ln\;P\left(s_{ij}|h_{ij}\right)+\overset{2N}{\underset{i=1}{\sum }}\ln\;P\left(h_{ij}\right). \end{array}$$

There is a data imbalance problem in the dataset; that is, the number of similar image pairs is much smaller than the number of nonsimilar image pairs in the image dataset. In this paper, a weight term *w*_*ij*_ is introduced to solve the data imbalance problem. Equation (2) can be further expressed as

(3) $$\begin{array}{c} \log\;P\left(H|S\right)\infty \;\log\;P\left(S|H\right)\log\;P\left(H\right)=\sum_{s_{ij}}w_{ij}\ln\;P\left(s_{ij}|h_{ij}\right)-\sum_{i=1}^{2N}\ln\;P\left(h_{ij}\right). \end{array}$$

As one of the system analysis methods, the gray correlation analysis method effectively avoids the problems of conventional mathematical and statistical methods and is widely used in various industries. Of course, in the process of application with the increase of its understanding, some algorithms with improved definitions of correlation factors were born. When performing the actual calculation, as far as possible, consider grading the influencing factors according to different levels and do not calculate the factors at different levels of the multilevel structure together. In this way, we can get an association tree represented by hierarchical tree structure, which is clear and intuitive, and can also carry out hierarchical analysis level by level.

(4) $$\begin{array}{c} X^{0}=\left(x_{i}^{0}\left(1\right),x_{1}^{1}\left(2\right),\ldots ,x_{i}^{n}\left(n\right)\right). \end{array}$$

Relying on statistical and probabilistic methods, the curve is first drawn based on statistical data, then the curve of the affiliation function is obtained from it, and finally, its mathematical expression is derived from that curve. Among them, the greater the number of fuzzy statistical experiments, the more stable the degree of affiliation. Although this method can specifically reflect the degree of “fuzzy” affiliation, there is a problem of large computational volume, as shown in Table 1.

Hard attention is given by filtering out valid regions and inputting them or, in the case of image research, by eliminating meaningless background data and then inputting them to effectively focus on objects in the target region by directly limiting the input content, but this method is not fully applicable in the field of time-series sequences, where even though there are differences in the importance of the input sequences, there is a certain amount of time-series relationship between the individual input sequences, and the sequences are in the same time series [16]. And under the same conditions, the prediction results of the experimental group increased by 1.84% compared with the ordinary decision tree algorithm, and the control group also increased by 2.91%. Under the same algorithm, the Light GBM algorithm achieves the smallest gap between the experimental group and the control group, indicating that it has good robustness and high value in football game prediction. Time-series relationship and the sequences are in different positions, so they cannot be explicitly located and removed. In addition, the hard attention mechanism is optimized by reinforcement learning, which is difficult to train and therefore relatively less general. The soft attention mechanism uses neural networks to train attention weights and weight global input features on space or channels to focus on specific spatial regions or channels, while the soft attention mechanism learns the attention network directly through end-to-end learning.

**Figure 1:**
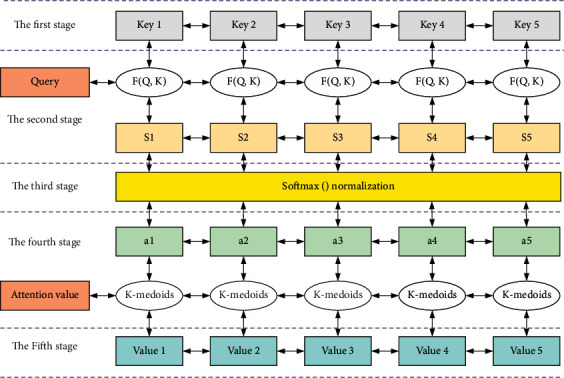
Schematic diagram of the principle of attention mechanism.

The weight coefficients are then normalized by the SoftMax function to obtain the weight coefficients, and finally, the weight coefficients and values are weighted and averaged to obtain the final representation of the classification features. The commonly used attention functions are additive attention and dot product attention functions. The attention function uses a forward neural network with a hidden layer to calculate the compatibility function, while the dot product attention function uses a highly optimized matrix multiplication, which can save memory resources and reduce computation, as shown in Figure 1.

In the first stage, different functions and computational mechanisms are introduced to calculate the similarity based on query and key. The commonly used methods are the dot product of vectors, the similarity of vector cosine, and the evaluation by introducing neural network. In this paper, we use the dot product, as shown in equation (5).

(5) $$\begin{array}{c} f\left(Q,K\right)=KQ^{T}. \end{array}$$

The second stage is to introduce a SoftMax-like calculation method to numerically transform the scores of the first stage to obtain the weight coefficients, which can be normalized to organize all the element weights into a probability distribution with a sum of 1 on the one hand and to highlight the weights of important elements through the inherent mechanism of SoftMax on the other hand, as shown in equation (6).

(6) $$\begin{array}{c} a_{i}=\text{soft}\max\left(f\left(f\left(Q,K\right)\right)\right). \end{array}$$

Data preprocessing, correlation analysis, and feature extraction should be performed on the original data before data mining. Data preprocessing is the most critical step in the whole data mining process, each step of data preprocessing affects the results of data analysis, and data analysis and feature extraction are the bridges connecting data mining algorithms, which also have an important impact on the data mining results. In this section, student data are mainly mined from the perspective of statistical features, clustering, and association rule analysis to explore the intrinsic relationship between student behavioral characteristics and student performance [17]. A grade prediction fusion model based on random forest, GBDT, and XGBoost is established. Finally, in view of the low prediction accuracy of the fusion model and the insufficient manual extraction of behavioral features, a CNN-LSTM score prediction model based on the attention mechanism is proposed. Finally, the findings mined from the two algorithms are combined to identify the main behavioral characteristics that affect student performance.

**Figure 2:**
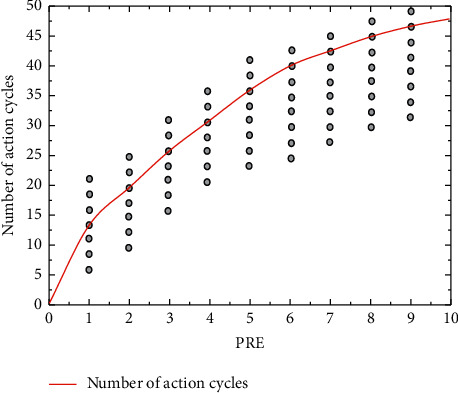
Action cycle number relationship.

Data integration is the process of integrating data from multiple data sources together to facilitate later data analysis and mining. Since the student data in this study come from data from different databases, including consumption data from the campus one-card system, borrowing data and access control data from the library management system, and student performance data from the academic affairs management system, the data coding format and attribute naming format in different databases are different. Therefore, before data integration, the naming of student data attributes of each data source should be unified. Since the student's school number has uniqueness, the student consumption data, student borrowing data, library access data, and academic achievement after data cleaning are associated by school number and integrated into one table. After data integration, it was found that some students' consumption information, borrowing information, and library access records had NULL values, and the NULL values were filled to 0 for future analysis. The data integrated after data integration was used as research data for student achievement prediction, which improved the efficiency of data mining tasks while solving the data inconsistency and redundancy problems, as shown in Figure 2.

The dots in Figure 2 represent the number of action cycles of the subjects corresponding to the specified RPE, and the dots of different subjects overlap. The correlation between the number of action cycles and RPE values was found to be positive (*P* < 0.01) at both speeds, with the correlation coefficients of r60°/s = 0.896 and r180°/s = 0.943. Since the angular velocity of the subject's movement was forced to be constant during the process, the number of movements × action cycles = movement time, so RPE was significantly positively correlated with movement time.

The core idea of neural network pruning, as one of the classical methods of model compression, is to subtract redundant parameters of the model while ensuring model accuracy through specific criteria. Neural network pruning is usually classified into structural pruning and unstructured pruning. Structural pruning refers to the complete subtraction of a recorder (convolutional kernel) from a convolutional neural network, which produces a large change in the structure of the network, so the accuracy of the model decreases greatly, but the model pruning rate is usually not high [18]. The normal operation of the digital and intelligent campus construction system in colleges and universities has accumulated a large amount of campus data, forming a big data environment in colleges and universities. Nonstructural pruning mainly refers to the operation by directly pruning the model parameters. The compression rate of nonstructural pruning is often higher than structural pruning, and the sparsity of the number of parameters in the network is high, but the actual number of parameters in the network is not reduced, and special hardware is needed to achieve the process of accelerated inference.

In the whole process, after determining the project content and purpose, the relevant data are collected and preprocessed, where the data preprocessing includes data selection, data cleaning, data integration, and data specification in four steps. Finally, the data mining results are obtained by using the relevant data mining models to mine the data set. The actual content of the project is related to getting the corresponding value of knowledge.

## 4. Sports Training Performance Prediction Model Design

This study focuses on predicting students' performance based on each student's behavioral characteristics, and the training set used in the process of designing the performance prediction model is the training set containing labels, that is, students' historical performance, so a supervised data mining classification algorithm is used to design and implement the performance prediction model. A classification model is a model that maps from discrete or continuous feature space to a discrete category being predicted. In this study, the feature space is the data of students' behavioral characteristics, and the predicted results are also the categories of students' final exam grades [19]. A supervised learning algorithm-based model is trained with an already existing training sample set to find the optimal prediction model that fits the data set and then uses this optimal prediction model to make predictions on the test set.

For supervised learning algorithms, each data point is labeled accordingly into category labels and numerical labels. This study is based on student behavioral characteristics to build the achievement prediction model, so it is necessary to design the student achievement prediction model using the classification algorithm model and the integration algorithm-based model from the supervised learning algorithm as the base learner.

In the process of designing the achievement prediction model, the selection of individual models is a crucial step in the whole student achievement prediction model design process. This section focuses on predicting students' future final grades by their student behavior during school, considering that there is a large individual variability in the data, and using a classification prediction algorithm to classify the sample indicators, which not only reduces the variability of the sample data but also reduces the complexity of the algorithm. In the selection of specific classification algorithms, this paper first selects the random forest algorithm, because the random forest algorithm has strong generalization performance and can effectively deal with the missing data when solving the classification problem in addition, the student achievement prediction classification is uneven, and the random forest can effectively balance the data set error.

**Figure 3:**
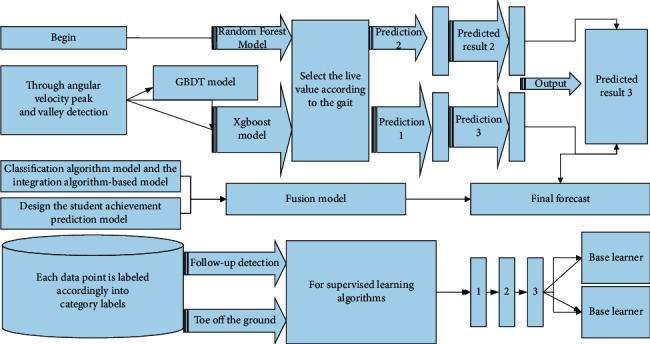
The process of building the achievement prediction model.

Secondly, the GBTC algorithm, which is particularly stable in classification efficiency, was subsequently selected for grade prediction, excluding the effect of neglected features in the feature extraction process. However, its disadvantage is that it only enhances static muscle strength and has significant angle specificity, which is poor in enhancing muscle endurance and has no significant help in improving the accuracy and coordination of exercise. However, in training, to achieve a full range of motion, the load can only be set at the minimum muscle torque. The fusion model construction process is shown in Figure 3.

The second time is to randomly select a subset of features and determine the best classification features by information gain and other indicators. Since there is no direct connection between each classification decision tree obtained during the construction of the random forest, when the constructed random forest model is input with new sample values again, this new set of sample values is input into each decision tree of the random forest, and these decision trees are judged separately on the feature values of these samples, from which the prediction value under this sample value is determined.

After the training data is input, the activation value of each neuron is calculated in the forward direction, then the reverse error calculation is performed, and the weights and bias gradients are obtained based on the errors. The weights and biases of the CNN interlayer connection are set up, and the gradient descent method is used to adjust the weights and biases to minimize the recognition error of fatigue in the application. The maximum number of network iterations is set to 10000. During the CNN training process, the loss curve is analyzed to determine whether the network converges, and the optimal fatigue recognition model is finally selected.

**Figure 4:**
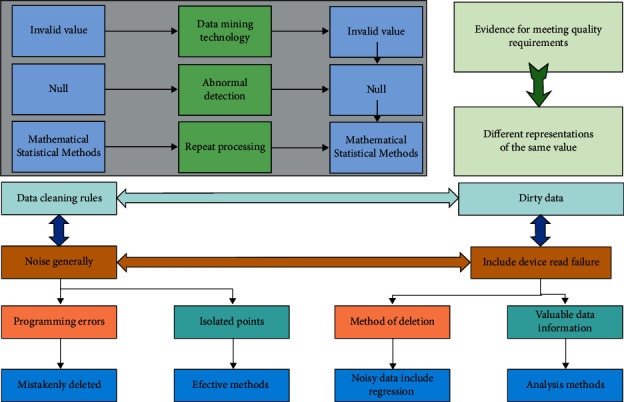
Data cleaning principle.

Since there is a large amount of missing, redundant, error, and noise data in the data, it is necessary to perform data cleansing on the data. In practice, after getting the data, we understand its basic situation and determine which data are unreasonable before carrying out the common data cleaning methods for cleaning. The projects usually include ball games, chess and cards, travel, and some popular national activities; mass sports are sports activities that are commonly held in real life when people want to improve their physical fitness, resist diseases, train candidate elites, and spend time. The data cleaning model is shown in Figure 4, which completes data streamlining, deweighting, and standard formatting.

The causes of noise generally include device read failure and programming errors. Such isolated points usually take the method of deletion, but the use of this method may also cause valuable data information to be mistakenly deleted. Therefore, more effective methods to deal with noisy data include regression, clustering, and other analysis methods. Common regression methods include linear regression and logistic regression. Linear regression is a mathematical-statistical method that establishes a dependence between the independent variable *x* and the dependent variable *y*. The data is smoothed by fitting the function to remove noise. Detection of outliers by clustering methods starts with identifying noisy data, aggregating all the data in the proximity to get the corresponding set, while the data outside the set is the object outlier [20]. Then, the values of the attributes on each clustering set can be determined to cause noise attributes and finally corrected by clustering determination.

Data normalization is a basic operation of data mining. Usually, in the process of data mining processing, different features in the data set have inconsistent magnitudes and excessive differences between values, which in turn affect the results of data processing. The above four models are trained with the achievement category as the category output, and the effectiveness of each classification model is tested utilizing fivefold cross-validation. To assess the effectiveness of the achievement prediction model, the accuracy, precision, recall, and *F*1 values are used. The specific operation of the fivefold cross-validation is to divide the behavioral feature set into five subsets, each time randomly select four subsets as the training set and the remaining one as the test set, and continuously change the training and test sets for five experiments; the result is the average of the results of the five experiments.

## 5. Generating the Results of the Adversarial Deep Neural Network Algorithm

**Figure 5:**
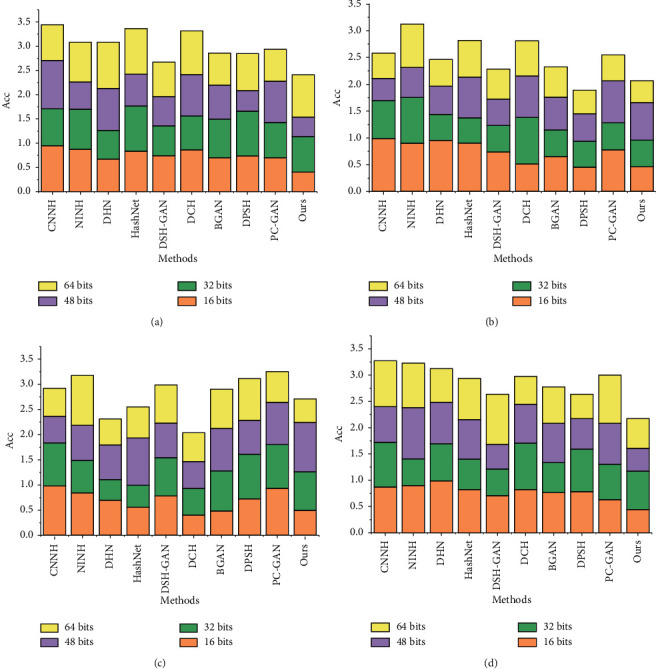
MAP values of the comparison algorithm on the benchmark dataset. (a) CIF AR-10, (b) MIRFlickr, (c) COCO, and (d) ours.

The MAP results of the compared algorithms are shown in Figure 5. The results show that the method based on point-pair supervised information outperforms the method based on pairwise supervised information, and the generative adversarial network-based hashing method outperforms the deep learning-based hashing method. Compared to DCH (deep hashing, pairwise supervised information), BH2I-GAN achieves an average increment of 1.3%, 4.3%, and 1.9% on the three datasets, respectively; compared to PC-GAN (hashing of GANs, pairwise supervised information), BH2I-GAN achieves an average increment of 6.8%, 7.6%, and 4.5%, respectively; compared to DSH-GAN (hashing of GANs, point-to-supervised information), BH2I-GAN achieves an average increment of 6.8%, 7.6%, and 4.5%, respectively; compared to DSH-GAN (hashing of GANs, point-to-supervised information), BH2I-GAN achieves 5.2%, 4.1%, and 4.6% average increments, respectively.

In image hash retrieval, P@H ≤ 2 is an important metric to evaluate the retrieval performance, which achieves a constant level of retrieval time and reflects the rapidity of hash retrieval. BH2I-GAN achieves a high P@H ≤ 2 retrieval result, indicating that BH2I-GAN achieves an efficient retrieval. As a result, the conclusions drawn from the analysis remain on the surface, and the full value of large amounts of data cannot be exerted. But the data and conclusions that administrators, teachers, and students really care about are precisely this hidden, less easily discovered, and valuable information. When the hash code becomes longer, the Hemming space becomes sparse and almost no data points fall into the Hemming sphere of radius 2 when the retrieval performance of most of the compared algorithms degrades.

Data integration is a way to ensure the logical or physical aggregation of data from different data sources, formats, and characteristics, thus providing comprehensive sharing for users. In this paper, a large amount of soccer data was obtained by cleaning the data above to obtain complete consistency and to ensure the sharing of data between this topic and the tutor; the data was unified and saved in a Mongo DB database according to data integration techniques.

The data consisted of the team's recent match odds and the team's recent match status data 65534 matches, the player's recent match status data 1168962 items, and the player transfer information table 535421 players transfer related information. Multiple database files are unified and integrated according to the team's name, team name, and match number as the primary key to generating the “soccer match history data analysis database.”

**Figure 6:**
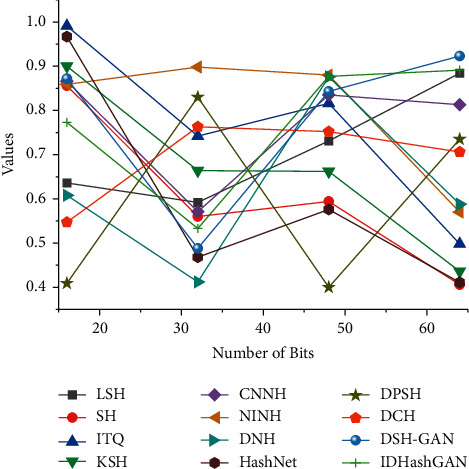
Comparison of hash retrieval algorithms.

Therefore, the deep hashing method based on deep learning and generative adversarial networks can also retrieve incomplete images. However, the retrieval accuracy is unacceptable when comparing the retrieval results of incomplete images, and some of the features learned by NINH, HashNet, DPSH, and DCH are not sufficient for hash coding, so the retrieval accuracy is low; DSH-GAN refers to incomplete images and uses GANs to generate images and labels, and the generated images have no reference value, resulting in low retrieval accuracy. MAP on the incomplete image dataset shows that the IDHashGAN algorithm can retrieve incomplete images efficiently. Compared with other algorithms, the average growth of MAP of IDHashGAN on noncomplete CIFAR-10, NUS-WIDE, and MIRFlickr datasets is 73.40%, 70.57%, and 70.33%, respectively, as shown in Figure 6.

The prediction accuracy of Light GBM is 73.29% after feature engineering, and the AUC value is 0.7976. Moreover, the prediction result of the experimental group is improved by 1.84% compared with the ordinary decision tree algorithm under the same conditions, and the control group is also improved by 2.91%. It is a quantitative comparison and comparison of the development situation and the comparison of the development situation. Finally, the relationship between the influencing factors of the multicharacteristic variables of the content is sought, and the changes in the dynamic motion can be grasped overall by discovering the main factors of the target. In the actual calculation, consider classifying the influencing factors according to different levels as far as possible. It is also found that the feature engineering method in this paper can effectively extract more valuable information about the game influencing factors after the actual comparison analysis. Therefore, it can be used to a certain extent to assist soccer match correlation analysis for soccer match prediction, improve the accuracy of soccer match prediction, and provide good data support for the following correlation analysis.

## 6. Analysis of Prediction Model Results

**Figure 7:**
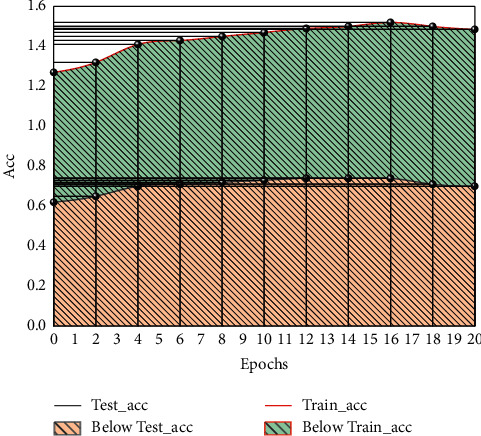
Accuracy curves of test set and training set.

To verify the effectiveness of the performance prediction model proposed in this section, the student behavior feature dataset in Figure 7 was used as the input value of the performance prediction model, and the predicted student performance categories were used as the output value of the model. Meanwhile, considering the sparsity of the data, 60% of the student behavior data were selected as the training data and 40% as the test data. The CNN model was used as the base classifier, and the stacked features from all weeks before the current week were used for the experiments. To assess the effectiveness of the achievement prediction model, various predictors were measured. Figure 7 shows the average accuracy, average precision, average recall, and average *F*1 value of student achievement prediction for different weeks tested with different models. The model accuracy in the training set increases with the number of training rounds, the model accuracy in the test set tends to decrease with the number of training rounds, and it can be learned that the model works best with 16 training rounds.

As the number of features per week increases, the accuracy of the stage grade prediction model tends to increase, and the prediction results of students' grades are at the peak in the second two weeks after the end of the course. In the first four weeks of the school year, students may be in an adaptive state, and their behavior changes a lot, so the accuracy of grade prediction is relatively low, while near the midterm or final, students face the pressure of exams, so their behavior is relatively regular, and the accuracy of grade prediction is relatively high. Therefore, it cannot be clearly located and removed. In addition, the hard attention mechanism is optimized through reinforcement learning, which is difficult to train and thus relatively less versatile. The CNN-LSTM model that learns the time-series data is more effective than the CNN model alone in predicting students' grades in consecutive weeks, and the model is relatively more effective; the CNN-LSTM model that introduces the attention mechanism outperforms the CNN-LSTM model that does not set weights in predicting students' grades.

**Figure 8:**
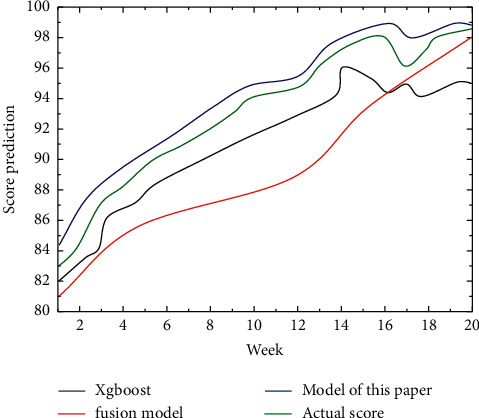
Comparison of the accuracy of XGBoost, fusion model, and the model in this section.

The performance of the CNN-LSTM model based on the attention mechanism is better than that of the established data mining-based model, and the accuracy of the CNN-LSTM model with the attention mechanism is significantly higher than that of the XGBoost and fusion models. After deep learning to automatically learn features, the model prediction accuracy was significantly improved, further indicating that there are shortcomings in the previous section using data mining techniques for grade prediction, not all student behaviors have a positive effect on student grade prediction, and some student behaviors may interfere with student grade prediction, as shown in Figure 8.

This study focuses on the prediction of student achievement based on student behavior, combining data mining classification algorithms and deep learning algorithms on student behavior data in campus activities. First, behavioral data such as consumption data, borrowing data, and library access control are analyzed through clustering and association rules to uncover the behavioral patterns hidden behind student behavior and the influencing factors related to student performance. Secondly, a fusion model based on random forest, GBDT, and XGBoost is developed for grade prediction. Finally, combined with the conclusions mined by the two algorithms, find out the main behavioral characteristics that affect students' grades.

The status of student behavior analysis and student achievement prediction at home and abroad is described, and the achievement prediction methods based on traditional machine learning are classified and summarized. By analyzing the current problems in constructing student achievement prediction models, the related research and applications of deep learning and sequence modeling are introduced. On this basis, the principles of machine learning classification algorithm, convolutional neural network, long- and short-term memory network, and attention mechanism are highlighted to provide theoretical support for the research of this paper.

## 7. Conclusion

Based on generative adversarial networks, this paper addresses the problems of unstable training, gradient disappearance, pattern collapse, and uncontrollable performance of discriminative networks caused by generative adversarial networks sampled in low-dimensional noise at the present stage and solves the above problems by changing the sampling method and introducing spectral normalization. Their motives, nonsimilarity/similarity, and advantages/disadvantages are analyzed, respectively. Then, generative adversarial networks and their derived models are specifically analyzed. The algorithm introduces the reconstruction network to construct a multigenerative adversarial network and uses reconstruction sampling to replace the low-dimensional noise sampling of the generative network. To ensure the consistency between the missing part of the restored image and the surrounding neighborhood part, the generative network receives not only the local missing part but also the neighborhood part of the missing part. To reduce the training time and memory consumption, the segmentation invariance principle is used to segment the neighborhood part into several segmented images of the size of the missing part. It is feasible to predict student grades from student behavior data, and the CNN-LSTM grade prediction model based on the attention mechanism has higher prediction accuracy and better performance than the data mining-based student grade prediction modeling approach, which verifies that the deep behavioral representation information extracted by using deep learning algorithms is more representative than the manually counted behavioral features and thus achieves better classification prediction results. In this paper, we use student behavior data as the original dataset, explore the relationship between student behavior and achievement, and verify the feasibility of student behavior to predict student achievement.

## Figures and Tables

**Table 1:** Learning algorithm steps.

1	For iter < I do:
2	*x* ← SA(X) {sample a batch of images from a set of real images X}
3	*x*′ ← *x*⊙*m* Add a random mask to each image in the batch of images
4	*f*iter < I_pre_ then:
5	*∂L*/*∂θ*_*g*_=∇_*θ*_*g*__1/*n*∑_*i*=1_^*n*^[*a* ln(1+*D*(*G*(*x*^*i*^, *m*^*i*^)))]
6	*θ*_*g*_ ← *θ*_*g*_+*lr*_1_*∂L*/*∂θ*_*g*_ Update the generative network
7	*X*′=*G*(*X*, *M*) Principal component network output reconstruction dataset X
8	Else:
9	Sampling a batch of images from the reconstructed image set and the real image set
10	*a*_*i*_=softmax(*f*(*f*(*Q*, *K*))) Update the discriminant network
11	*lr*_1_ *∂L*/*∂θ*_*g*_=∇_*θ*_*g*__1/*n*∑_*i*=1_^*n*^[ln(1+*D*(*G*(*x*^*i*^, *m*^*i*^)))] Calculate the parameter gradient
12	*x*′↔*x*⊙*m* Update the hash network
13	End if
14	End for

## Data Availability

The data used to support the findings of this study are available from the corresponding author upon request.
